# The effect of using a chatbot integrated with the ARCS motivation model in physiology education: a mixed-methods study

**DOI:** 10.3389/fphys.2026.1816470

**Published:** 2026-05-28

**Authors:** Yasin Ali Cimen, Gunes Bolatli

**Affiliations:** 1 Department of Physiology, Faculty of Medicine, Yalova University, Yalova, Türkiye; 2 Department of Anatomy, Faculty of Medicine, Yalova University, Yalova, Türkiye

**Keywords:** academic achievement, ARCS motivation model, chatbot-assisted learning, physiology education, technology acceptance

## Abstract

**Introduction:**

This study examined the effects of integrating the ARCS (Attention, Relevance, Confidence, and Satisfaction) Model of Motivational Design with an AI-supported chatbot on academic achievement and learner perceptions in physiology education.

**Methods:**

The study was conducted with 75 students enrolled in university-level physiology courses. The control group (Traditional) received instruction using traditional teaching methods, while the teaching process for the second group (ARCS) was structured according to the ARCS model. The third group used a chatbot-supported learning environment in addition to the ARCS model (ARCS +Chatbot). Academic achievement and cognitive load were assessed using pretest and post-test measurements in all groups, while technology acceptance was measured only in the ARCS+Chatbot group using the Technology Acceptance Model (TAM) before and after implementation. Additionally, to examine the chatbot-supported learning experience in greater depth, a semi-structured interview form was administered to students in this group, and the data were analyzed using thematic analysis.

**Results:**

Quantitative findings indicated that academic achievement increased significantly across all groups from the pre-test to the post-test (p<.001). However, the increase in achievement differed across groups, with the ARCS+Chatbot group performing significantly better than both the traditional instruction group (p<.001) and the ARCS group (p<.01) on the posttest. In contrast, no significant differences were found in pre-test and post-test measurements of cognitive load or in between-group comparisons (p>.05). TAM analyses revealed that chatbot use significantly increased perceived usefulness (p<.05) but did not result in a significant change in perceived ease of use (p>.05). Qualitative findings indicated that students tend to perceive the chatbot as a tool that supports their learning process and complements the instructor’s role.

**Discussion:**

The integration of an AI-supported chatbot into the ARCS model improved academic achievement without increasing cognitive load, suggesting its potential as an effective instructional approach in physiology education.

## 1 Introduction

There is broad consensus that basic sciences are critically important in medical education and form the indispensable foundation of clinical practice (Finnerty et al., 2010). Basic science knowledge forms the foundation of clinical reasoning, helping students develop the thinking skills necessary for effective decision-making in medical practice (Csaba et al., 2025). Medical educators are concerned that clinicians use their basic science knowledge to a limited extent in clinical practice, resulting in a decline in the retention of this knowledge after the early years of medical education (De Bruin et al., 2005. Information that is not supported by repetition and meaningful use is not sustainable in the long term; studies show that approximately 50–60% of this type of information is lost within two years and that forgetting increases over time (Taveira-Gomes et al., 2015). The physiology course is one of the fundamental courses in medical education that enables students to understand the normal functioning of the human body (Hooi and Koh, 2005). Physiology is an area that can be more easily transferred to clinical practice compared to other basic sciences included in medical education, and a significant portion of the clinical questions asked during the early clinical training period are based on physiological reasoning (Hadad et al., 2025). However, there are also findings that suggest physiology knowledge is not sufficiently preserved during clinical training; indeed, it has been reported that only 26% of 204 medical interns scored 60% or higher on a physiology knowledge test (AlMohanna et al., 2018). Therefore, the permanent learning of physiological concepts and mechanisms is critical for students to develop accurate and sustainable clinical reasoning skills from the early stages of clinical education.

Pre-clinical medical education is structured to expect students to acquire a broad knowledge base in physiology in a short period of time and to reinforce this knowledge intensively (Lazić et al., 2006). However, the abstract nature of physiological mechanisms and the interrelatedness of numerous concepts can make it difficult for students to understand this course and retain the information they learn. Some studies provide solid evidence of this difficulty. J. Michael et al. identified three main factors contributing to the difficulty of physiology: the nature of the discipline, teaching methods, and students’ approaches to learning (Michael, 2007). Kay Colthorpe et al. noted that students found it difficult due to their lack of familiarity with the subject and the level of detail required (Colthorpe et al., 2018). The fact that physiological concepts often cannot be considered in isolation and require integration across different systems necessitates a significant degree of cognitive flexibility in the learning process (Lira and Gardner, 2017). The conceptual density and mechanism-based structure of physiology can lay the groundwork for an increase in cognitive load during the learning process. Cognitive Load Theory explains the load generated during the learning process through three components: internal, external, and related (Van Merriënboer and Sweller, 2010). Research conducted over more than thirty years has shown that when these loads exceed working memory capacity, learning is inevitably impaired (Ginns and Leppink, 2019). While the intrinsic load arising from the nature of the content to be learned cannot be directly altered, the total cognitive load can be reduced by using appropriate teaching methods that target the extrinsic load associated with instructional design (Young et al., 2014). In this context, teaching approaches that address the relationships between physiological processes in a holistic manner can contribute to keeping cognitive demands at a manageable level during the learning process and facilitate the meaningful structuring of knowledge by supporting student participation in class.

In order for the transfer of learning to occur, teachers must consciously structure their teaching processes in a way that supports this transfer (Anderson and Beavis, 2020). Although the transfer of learning is one of the fundamental goals of formal education, achieving this in practice is often difficult (Michael, 2022). Given these difficulties in knowledge transfer, it has been reported that traditional lecture-centered teaching approaches are associated with lower academic achievement compared to methods that encourage active participation (Freeman et al., 2014). It has been reported that interactive learning techniques increase student achievement and lead to significant improvement, particularly among low-performing students (Gauci et al., 2009). Motivation is a fundamental factor that supports the continuity of the learning process and is directly related to academic achievement (Paas et al., 2005). For this reason, the need for teaching approaches that focus on active student participation and motivation to learn in physiology education is becoming increasingly apparent.

The widely used ARCS Model of Motivational Design (Attention, Relevance, Confidence, and Satisfaction) provides a systematic framework for incorporating motivational strategies into instructional design (Keller, 1987). Furthermore, it is argued that many strategies aimed at increasing student motivation align with the core components of the ARCS model (Hodges, 2004). The effectiveness of the ARCS model to the learning process is supported by research conducted in various health science disciplines. A study conducted on nursing students reported that ARCS model-based interactive digital learning materials support learning motivation, self-efficacy, and clinical skill development (Chen et al., 2025). Another ARCS model-based study involving biology students enrolled in an animal physiology course highlighted the importance of adapting instructional strategies to better support student motivation (Amin et al., 2016). However, studies on the effects of this approach on learning outcomes in the context of human physiology education are limited.

It is well known that students’ learning preferences have changed in recent times and that they have embraced technology as a natural part of their learning processes (Owolabi and Bekele, 2021). Therefore, educators must take advantage of the opportunities offered by technology to create learning environments suited to the changing student profile (Bolatli and Kizil, 2022). With the widespread adoption of digital technologies in education, new tools supporting motivation-based instructional design have also begun to be used in recent years (Yu, 2024). Artificial intelligence is a technology that enables machines to mimic human-like intelligence, targeting fundamental cognitive abilities such as learning, problem-solving, reasoning, and pattern recognition (Fitria, 2021; Shrivastava et al., 2024). In recent years, AI-supported chatbots (chatbots) have been increasingly used in educational settings due to their ability to interact with students, provide immediate responses, and offer personalized learning support (Singh and Namin, 2025; Leng, 2024). These systems can extend learning beyond classroom hours and support knowledge consolidation (Boscardin et al., 2024). Gemini, a versatile multimodal chatbot platform, has attracted attention in educational settings due to its ability to process various types of data and enhance response accuracy through human-feedback-based learning approaches (Wang et al., 2017; Yigci et al., 2025). However, despite the growing use of chatbot-supported learning environments in education, empirical evidence regarding their instructional effectiveness in human physiology education remains limited.

While interest in motivation-based instructional design is growing and the use of chatbot-supported learning environments in health sciences education is becoming increasingly widespread, empirical evidence demonstrating the combined effect of these two approaches in the context of physiology education remains limited. In particular, the effects of integrating the ARCS model with chatbot-supported learning environments on university students’ academic achievement, cognitive load levels, and perceptions of technology acceptance in physiology education have not been sufficiently investigated.

## 2 Research questions

Physiology is a subject that forms both the conceptual basis of medical education and the transition to clinical practice. It must be taught through effective and lasting learning to ensure professional competence after graduation (Ghosh, 2007). Traditional teaching and assessment approaches do not sufficiently support the learning process due to their teacher-centered structures and limited student participation (Nybo and May, 2015; Diwan et al., 2017). The use of artificial intelligence technologies in health sciences education offers significant potential in terms of supporting and personalizing learning processes (Bolatli et al., 2026; Bayne, 2015).

Therefore, it is hypothesized that the instructional approach based on the ARCS model and its integration with a chatbot-supported learning environment may enhance academic achievement compared to traditional teaching methods and support the learning process without negatively affecting cognitive load levels. In addition, the findings may provide valuable insights into technology acceptance and students’ perceptions in ARCS-based chatbot-supported learning environments.

In line with these hypotheses, the study sought answers to the following research questions:

1. Do traditional teaching methods, ARCS model-based instruction, and ARCS model-based chatbot-supported instruction lead to significant differences in academic achievement?
2. Do these instructional approaches affect students’ perceived cognitive load during the learning process?
3. Is there a significant relationship between academic achievement, students’ technology acceptance, and their perceptions of the learning experience in ARCS model-based chatbot-supported learning environments?

## 3 Method

A convergent mixed-methods research design was adopted in this study, in which quantitative and qualitative data were collected concurrently and integrated during the analysis phase. Within this framework, the study examined the effects of ARCS-model-based instruction and its integration with an additional chatbot-supported learning environment on learning outcomes in human physiology education. Quantitative data were used to assess academic achievement, perceived cognitive load, and technology acceptance levels, while qualitative data were collected to gain a deeper understanding of students’ experiences with the chatbot-supported learning process.

### 3.1 Research design

This study is quasi-experimental research designed to examine the effects of different learning approaches on learning outcomes in teaching physiology to nursing students. The study employed a research design that utilized pre-test and post-test measurements, enabling intergroup comparisons. Seventy-five first-year nursing students and students who signed the voluntary participation consent form were included in the study. Students who graduated from secondary schools specializing in health sciences and nursing students who failed their physiology course in previous years were not included in the study. Ethical committee approval has been obtained from the Yalova University Health Sciences Non-Interventional Clinical Research Ethics Committee (no: 2025/469).

### 3.2 Participants and group structure

**Figure 1:**
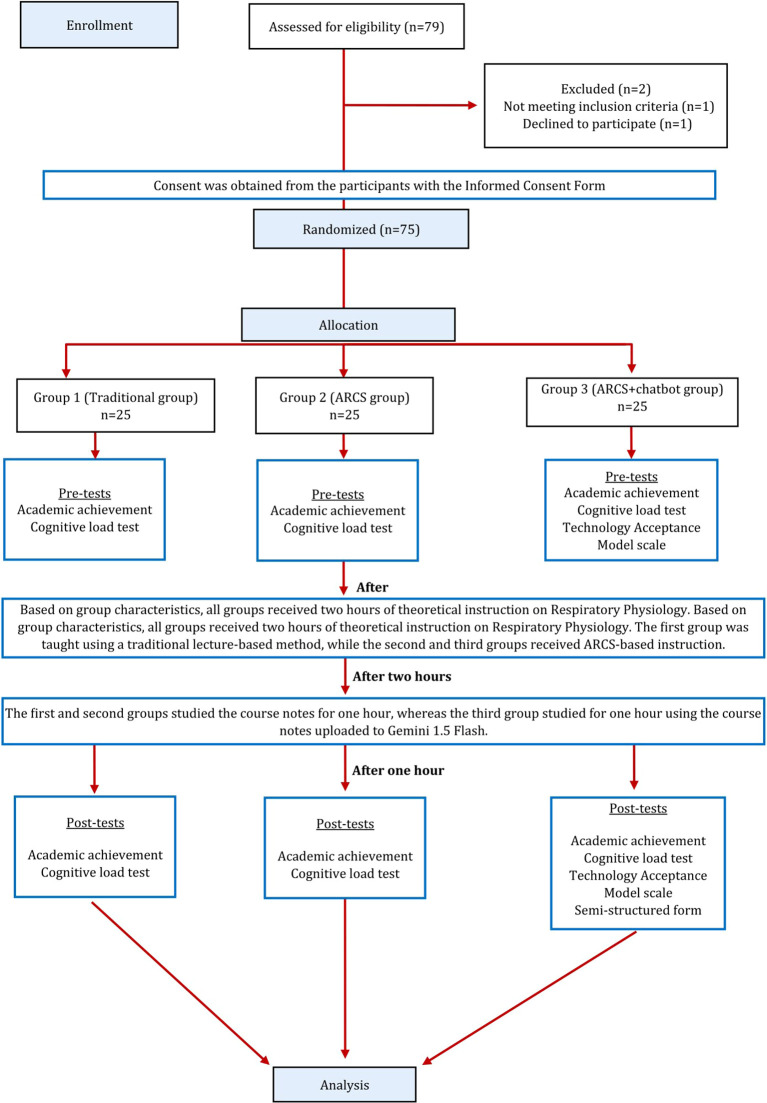
Study design and procedure flow diagram. Flow chart showing the study design and implementation process. Participant eligibility assessment, randomization, group assignments, teaching applications, and pre-test–post-test–follow-up measurement times are presented.

The students participating in the study (n = 75) were randomly assigned to three groups according to the learning approach used. The first group (Traditional, n = 25) used standard lecture notes prepared using the traditional method. The second group (ARCS, n = 25) used lecture notes structured based on the ARCS model. The third group (ARCS+Chatbot, n = 25), in addition to ARCS model-based lecture notes, benefited from an interactive learning environment through an artificial intelligence–supported chatbot. All groups were organized to have equivalent conditions in terms of course content and teaching duration. Participants were randomly assigned to three groups using a computer-based randomization procedure. A class list was entered into a digital system, and group allocation was performed using a random assignment algorithm to ensure unbiased distribution. This approach was used to minimize selection bias and increase the likelihood of baseline comparability among groups. To minimize potential interaction between groups, the sessions were conducted in separate classrooms. Consistency in the teaching process was ensured by having the same instructor teach all groups. Instructional fidelity was maintained through standardized lesson plans. The flow of participants through the study and the timing of measurements are presented in **Figure 1**.

### 3.3 Application steps by group

The study began with a two-hour theoretical lecture on respiratory physiology given to all groups after the appropriate scales were applied. Respiratory physiology, which includes conceptually complex mechanisms such as gas exchange, ventilation-perfusion relationships, and partial pressure dynamics, provides a suitable context for examining the effects of cognitive load and motivational instructional design on the learning process. The traditional group received a presentation using traditional lecture notes, while the ARCS and ARCS+Chatbot groups received a presentation using lecture notes prepared according to motivational design principles. Following this lecture, students were given a one-hour individual study opportunity tailored to the characteristics of their own group. During this process, the traditional group worked only with traditional lecture notes, while the ARCS group worked with lecture notes prepared according to motivational design principles; students in the ARCS+Chatbot group attempted to understand the lecture by transferring the lecture notes prepared according to motivational design principles to a chatbot-supported learning environment. All chatbot interactions and academic achievement tests were conducted in Turkish.

Traditional group: After administering a preliminary academic test and a preliminary cognitive load test, a 2-hour theoretical lecture on respiratory physiology was delivered using slides prepared with traditional lecture notes. After the theoretical lecture, students were given one hour to study the lecture notes. After one hour of free study, the post academic test and post cognitive load test were administered.

ARCS group: After administering a pre-academic test and a pre-cognitive load test, a 2-hour theoretical lecture on respiratory physiology was delivered using slides prepared with ARCS-based lecture notes. The teaching process in ARCS model-based teaching was designed to target the components of attention, relevance, confidence, and satisfaction through attention-grabbing questions, clinical context linking, structured learning tasks, formative feedback, short videos, and GIFs. After the theoretical lecture, students were given one hour to study the lecture notes. Following the one-hour free study period, the post academic test and post cognitive load test were administered.

ARCS+Chatbot group: After administering the academic achievement pre-test, cognitive load pre-test, and Technology Acceptance Model (TAM) pre-test scale, a 2-hour theoretical lecture on respiratory physiology was delivered using slides prepared based on ARCS principles. The instructional process was designed to address the ARCS components through attention-grabbing questions, clinical context integration, structured learning tasks, formative feedback, short videos, and GIFs. Following the theoretical lecture, students were given one hour for self-study. During this period, students interacted with an AI-supported chatbot (Gemini 1.5 Flash) by uploading their ARCS model-based lecture notes and instructing the chatbot to “review these lecture notes with me.” After the 1-hour study period, the academic achievement post-test, cognitive load post-test, TAM post-test scale, and a semi-structured interview form were administered.

### 3.4 Data collection process

All students participating in the study provided written informed consent. Prior to the intervention, all participants completed the academic achievement pre-test and the cognitive load scale to assess their baseline knowledge and perceived mental effort. Students in the ARCS+Chatbot group also took the TAM scale to determine their acceptance of the technology used in chatbots. During the intervention, the traditional group received a two-hour theoretical lecture on respiratory physiology using standard lecture notes, while the other two groups received a two-hour lecture using structured course materials according to the ARCS model. Following the lecture, all students were given one hour of individual study time. After the completion of the study session, the traditional and ARCS groups were re-administered with the post academic achievement test and the post cognitive load scale. In the ARCS+Chatbot group, in addition to these measurements, the post TAM scale was administered, and semi-structured interview forms were conducted to evaluate students’ chatbot-assisted learning experiences in detail. The analyses were conducted on participants whose data for the relevant measurements was complete.

### 3.5 Characteristics of the scales used

Academic achievement test: It consists of a total of 20 questions; 18 of these are multiple-choice questions with five options, one is a fill-in-the-blank question, and one is a figure-based question. The test was administered to all students at the beginning (pre-test) and end (post-test) of the study (Appendix **Figure 1**). The test items were scored dichotomously (correct/incorrect), and the internal consistency of the academic achievement test was evaluated using the Kuder–Richardson coefficients (KR-20 and KR-21). The reliability analysis was conducted using the post-test data obtained at the end of the study. The KR-20 coefficient, which is considered to yield more appropriate results in tests where item difficulties are not homogeneous, was used as the primary reliability measure, while the KR-21 coefficient was reported as a supplementary indicator. A KR-20 coefficient of.70 or higher indicates that the test possesses an acceptable level of internal consistency (Kuder and Richardson, 1937).

Cognitive load test: Consists of a single question. The cognitive load expended by students in understanding the physiology course was tested on a scale from 1 to 9 (Paas and Van Merriënboer, 1993). This measurement is based on the single-item self-report approach commonly used in educational research. It was administered to all students twice, at the beginning and end. The responses given by students before and after the application were compared (**Supplementary Figure 2**).

Technology Acceptance Model scale: It consists of 28 questions in total, with 14 questions aimed at determining perceived usefulness and 14 questions aimed at determining perceived ease of use. Students rated each question as 1) Strongly Disagree, 2) Disagree, 3) Not Sure, 4) Agree, 5) Strongly Agree. It was administered only to the ARCS+Chatbot group, twice in total: before and after. Responses to the same question given before and after were compared. The TAM scale used in this study was adapted from the original TAM framework developed by Davis (Davis, 1989), using the scale form developed by Tubaishat (Tubaishat, 2018) for health and education contexts. The Turkish adaptation of the scale was made by Parlak (Parlak, 2019). In line with the objectives of this study, the item statements were adapted to reflect the chatbot-supported learning environment and students’ interactions with the chatbot. The adapted items were reviewed by the research team to ensure clarity and contextual appropriateness. No formal pilot testing was conducted prior to the implementation of the adapted items. Although the construct validity of the scale had been established previously, internal consistency was re-evaluated in the current sample (**Supplementary Figure 3**).

Semi-structured interview form: It was applied only once as the scale to the ARCS+Chatbot group. Data was collected through four open-ended questions designed to reveal students’ thoughts on the use of chatbots in the learning process. The questions focused on four main areas: (1) perceived effects on learning and comprehension, (2) learning motivation, (3) difficulties and limitations encountered during use, and (4) perceived effect on student-teacher interaction. Participants provided written responses, which were included in the analysis without modification. All responses were anonymized prior to analysis. A thematic analysis was applied to the students’ responses. This qualitative approach allows for an in-depth examination of participants’ perceptions, interpretations, and experiences rather than quantifying predefined outcomes (**Supplementary Figure 4**).

### 3.6 Statistical analysis

The analysis of the data obtained from the research was performed using SPSS 20.0 software. Graphs were created using GraphPad Prism 8 (GraphPad Software, USA). The analysis process consisted of the following stages:

Sample Size and Power Analysis: Based on the parameters reported in the reference study (mean difference = 2.5, standard deviation = 3.10), a sample size calculation indicated that at least 24 participants per group are required to achieve 80% statistical power at a significance level of α = .05. Accordingly, 25 participants were included in each group (Joseph et al., 2026). Testing Normality and Assumptions: The normality of the data distribution was examined using the Kolmogorov-Smirnov and Shapiro-Wilk tests; it was determined that the data followed a normal distribution. Accordingly, it was decided to use parametric tests in the analysis. Reliability Analyses: The internal consistency of the Academic Achievement Test was assessed using Kuder-Richardson 20 (KR-20) and Kuder-Richardson 21 (KR-21). The internal consistency of the TAM Scale and its subscales (Perceived Usefulness and Perceived Ease of Use) was assessed using Cronbach’s alpha coefficients. Quantitative Data Analysis: A 3 (Group: Traditional, ARCS, ARCS+Chatbot) × 2 (Time: Pre-test, Post-test) mixed-design ANOVA was performed to examine changes in academic achievement and cognitive load over time and to determine whether these changes differed across groups. Following the identification of significant interaction effects, Tukey HSD *post-hoc* tests were conducted for pairwise comparisons. In addition, a paired samples t-test was conducted to examine the pre-test and post-test changes in technology acceptance perceptions of the experimental group (ARCS+Chatbot). Thematic Analysis: The analysis of qualitative data was conducted within an inductive framework, based on Braun and Clarke’s thematic analysis approach, without using any pre-determined themes (Braun and Clarke, 2006). In the first stage of the analysis process, all participant responses were read multiple times to gain a comprehensive familiarity with the dataset. Meaningful expressions were manually coded, and the coding process was conducted independently from the outset by two researchers. Positive and negative opinions were included in the analysis in a balanced manner. The coding process was performed without the use of any qualitative analysis software; then, the researchers compared their codes, and any differences were discussed and resolved through consensus. The resulting codes were grouped according to their similarities to creating main themes and sub-themes. The resulting theme structure was evaluated in terms of inter-theme consistency, scope, and conceptual clarity, paying particular attention to the integrity of the codes within the themes and the distinguishability of the themes from each other. In the final stage of the analysis, the themes were compared again with the dataset to check content consistency and were clearly defined and labeled. Considering that participants may express opinions under more than one theme, the number of participants (n) contributing to each theme was reported.

## 4 Results

### 4.1 Reliability analysis of the achievement test

The internal consistency reliability of the achievement test was assessed using the KR-20 and KR-21 coefficients. The analysis revealed a KR-20 coefficient of.80 and a KR-21 coefficient of.75. The findings indicate that the achievement test possesses an acceptable level of internal consistency (**Table 1**).

**Table 1:** Internal consistency reliability coefficients of the academic achievement test.

Test	Number of items	KR-20	KR-21
Academic Achievement Test	20	.80	.75

The KR-20 and KR-21 coefficients indicate the internal consistency reliability of the test.

### 4.2 Effects of instructional designs on academic achievement and cognitive load

#### 4.2.1 Comparison of pre-test and post-test achievement scores by group

**Figure 2:**
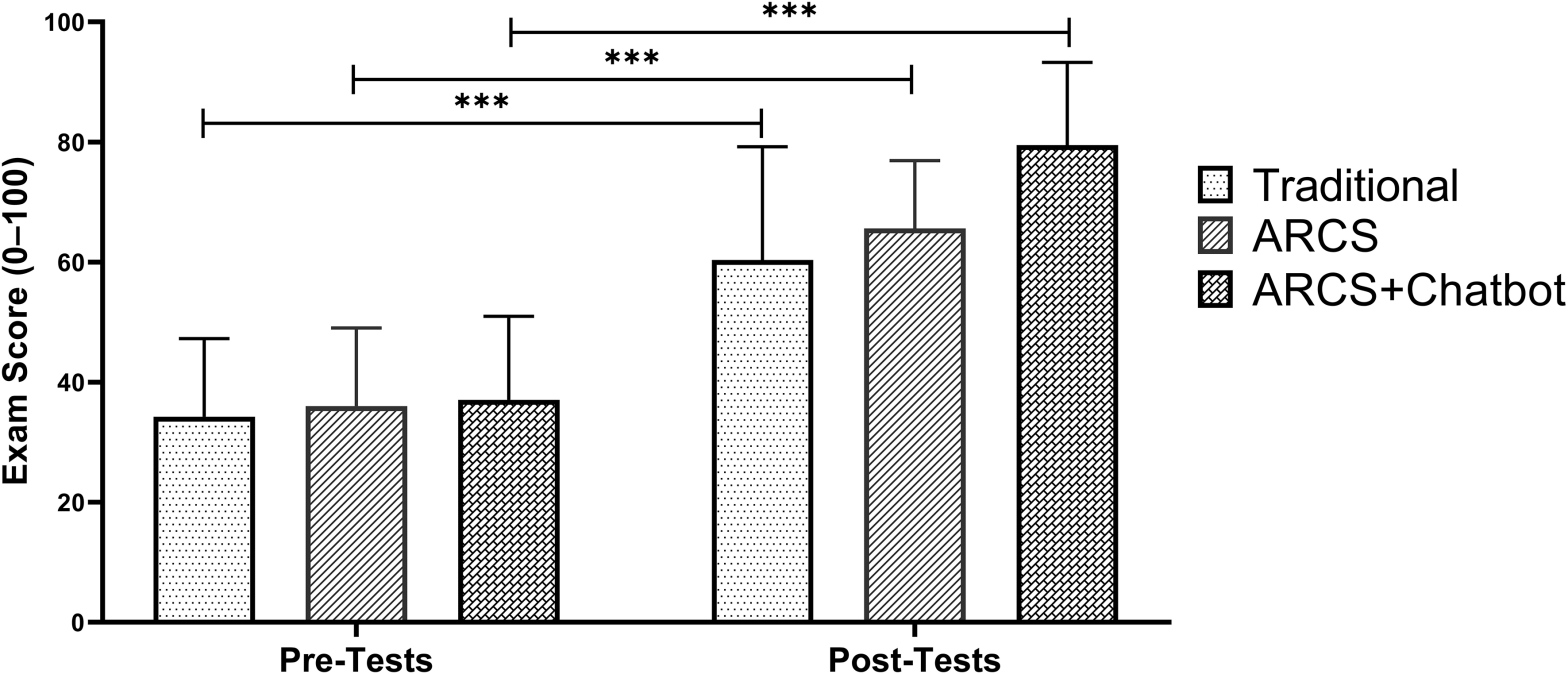
Pre-test and post-test exam scores across study groups. Pre-test and post-test exam scores across study groups. Bars represent mean ± standard deviation (SD) values. Group and time effects were examined using a two-way mixed-design ANOVA. Statistical significance is indicated as ***p <.001. Traditional = traditional instruction group; ARCS = ARCS-based instruction group; ARCS+Chatbot = ARCS-based instruction supported with chatbot.

**Figure 3:**
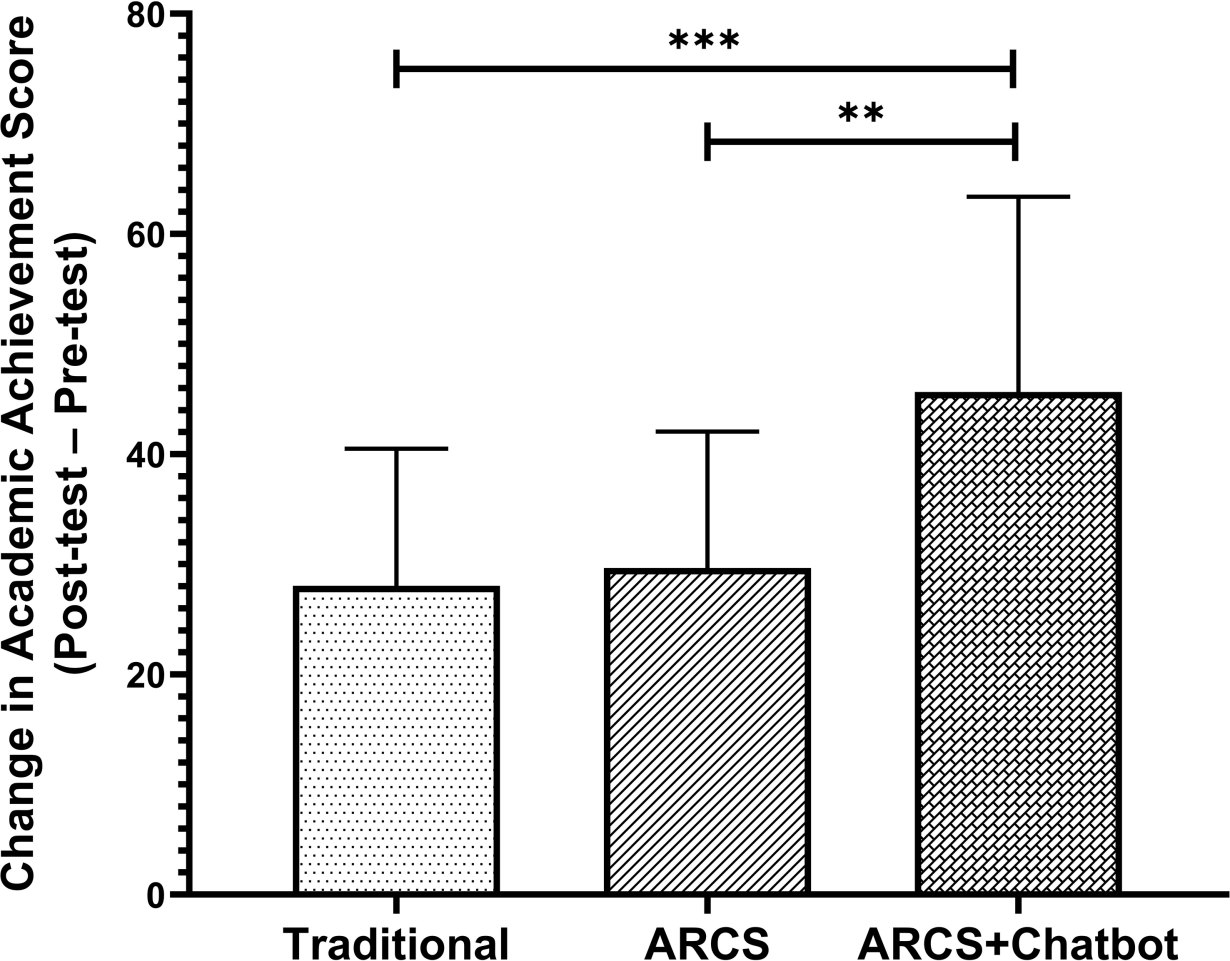
Change in academic achievement scores from pre-test to post-test across study groups. Bars represent mean ± standard deviation (SD) values. Group comparisons are based on Tukey HSD *post-hoc* tests. Statistical significance is indicated as **p <.01 and ***p <.001. Traditional = traditional instruction group; ARCS = ARCS-based instruction group; ARCS+Chatbot = ARCS-based instruction supported with chatbot.

There was no significant difference in pre-test academic achievement scores between groups (p >.05). It was determined that post-test academic achievement scores increased significantly in all groups compared to pre-test measurements (p <.001; **Figure 2**). To examine whether the change in academic achievement differed across groups, a 3 (Group: Traditional, ARCS, ARCS+Chatbot) × 2 (Measurement: Pre-test, Post-test) mixed design repeated measures ANOVA analysis was applied. The analysis results showed that academic achievement scores increased significantly from the pre-test to the post-test (F(1, 65) = 387.50, p <.001, partial η² = .86). Furthermore, it was determined that the increase in academic achievement varied across groups (Measure × Group interaction: F(2, 65) = 10.33, p <.001, partial η² = .24). The Tukey HSD multiple comparison test conducted to determine the source of the significant interaction revealed that there was no significant difference in academic achievement increase between the Traditional group and the ARCS group (p = .469). In contrast, the academic achievement increase of the ARCS+Chatbot group was significantly higher than both the Traditional group (p <.001) and the ARCS group (p <.001) (**Figure 3**).

Effect size analyses show that the ARCS+Chatbot group has a high level of effect compared to both the Traditional group (d = 1.17) and the ARCS group (d = .99). In contrast, the difference between the Traditional and ARCS groups was found to be low (d = .18).

#### 4.2.2 Comparison of pre-test and post-test cognitive load scores across groups

**Figure 4:**
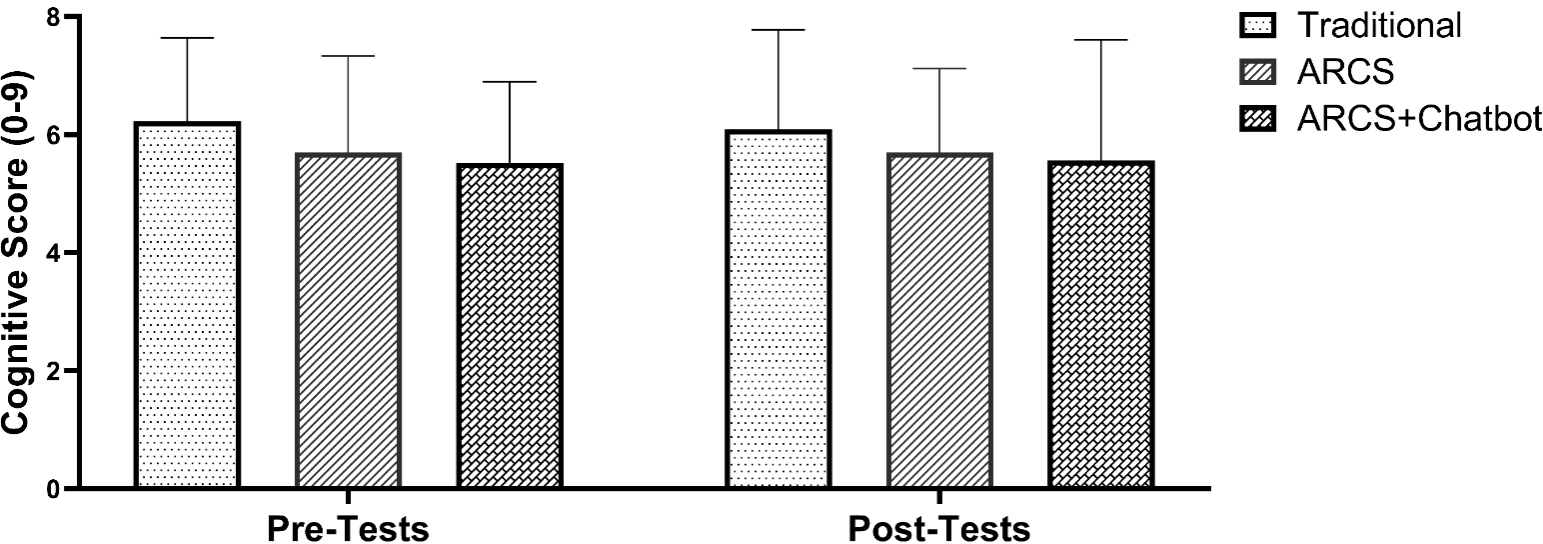
Pre-test and post-test cognitive load scores across study groups. Bars represent mean ± standard deviation (SD) values. Group and time effects were examined using a two-way mixed-design ANOVA. Traditional = traditional instruction group; ARCS = ARCS-based instruction group; ARCS+Chatbot = ARCS-based instruction supported with chatbot.

To examine whether cognitive load scores changed across measurements and whether this change differed across groups, a 3 (Group) × 2 (Measurement: Pre-test, Post-test) mixed-design repeated measures ANOVA analysis was conducted. The analysis results revealed that cognitive load levels did not show a significant change between the pre-test and post-test measurements (*F*(1, 65) = .025, *p* = .874, partial η² <.001; **Figure 4**). Furthermore, it was determined that the change in cognitive load did not differ across groups (Measure × Group interaction: *F*(2, 65) = .076, *p* = .927, partial η² = .002). Cohen’s d indicated trivial changes in the Traditional (d = .09), ARCS (d = .00), and ARCS+Chatbot (d = .02) groups.

### 4.3 Results of the technology acceptance model scale

#### 4.3.1 Reliability results of the technology acceptance model scale

The reliability of the scale in the current sample was assessed using Cronbach’s Alpha internal consistency coefficient. The analysis results showed that α = .93 for the Perceived Usefulness sub-dimension, α = .84 for the Perceived Ease of Use sub-dimension, and α = .82 for the entire scale (**Table 2**).

**Table 2:** Internal consistency coefficients for the perceived usefulness and perceived ease of use scale.

Subscale	Number of items	Cronbach’s alpha (α)
Perceived Usefulness	14	.93
Perceived Ease of Use	14	.84
Total Scale	28	.82

#### 4.3.2 Changes in technology acceptance perceptions of the ARCS+Chatbot group

To examine changes in participants’ perceptions of technology within the ARCS+Chatbot group, pre- and post-intervention TAM scores were compared using a paired t-test (**Table 3**). The analysis results show that the post-test scores in the Perceived Usefulness dimension increased at a statistically significant level compared to the pre-test scores (p = .038). In contrast, no statistically significant difference was found between the pre-test and post-test scores in the Perceived Ease of Use dimension (p = .776).

**Table 3:** Comparison of pre-test and post-test scores for the technology acceptance model in the ARCS+Chatbot group.

TAM		Mean	SD	P
Perceived Usefulness	Pre-test	3.68	0.49	.038
	Post- test	3.92	0.58	
Perceived Ease of Use	Pre-test	2.94	0.43	.776
	Post- test	2.96	0.46	

Mean, Average; SD, Standard deviation. p-values indicate the results of the dependent groups t-test comparing pre-test and post-test scores.

### 4.4 Students’ views on chatbot-supported learning

**Figure 5:**
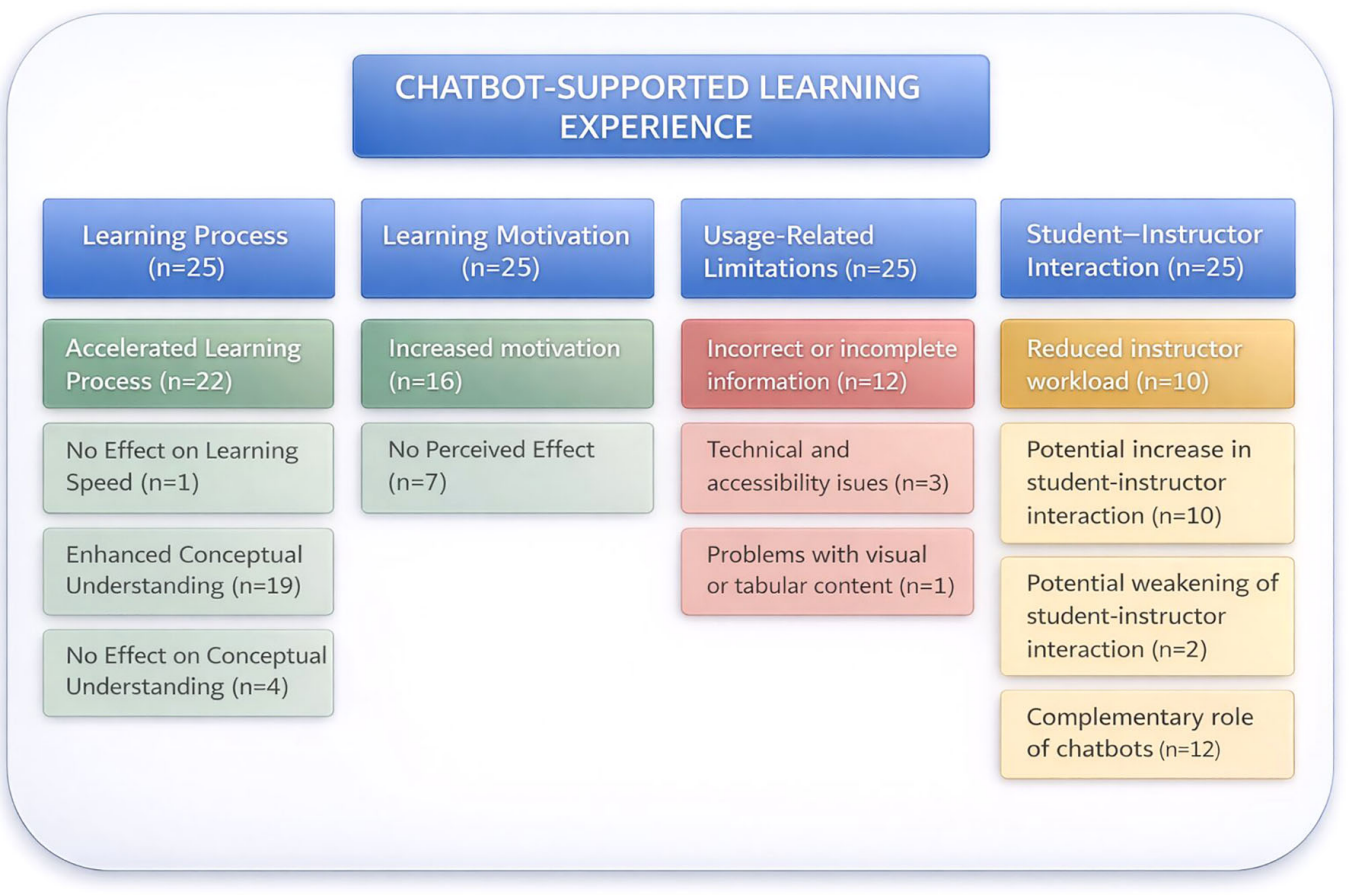
Results of the thematic analysis regarding the chatbot-supported learning experience in the ARCS+Chatbot group. This figure shows the results of the thematic analysis of qualitative data obtained from semi-structured interviews conducted with students in the ARCS+Chatbot group. The analysis grouped the chatbot-supported learning experience into four main themes: learning process, learning motivation, limitations of use, and student-instructor interaction. The values indicated next to each sub-theme show how many participants expressed the relevant opinion.

Thematic analysis of data obtained from semi-structured interviews conducted in the ARCS+Chatbot group shows that the chatbot-supported learning experience revolves around three main themes. Learning Process and Motivation: A large portion of participants indicated that using the chatbot increased their learning speed and quality. Under this theme, it was determined that the learning process accelerated (n=22), conceptual understanding deepened (n=17), and overall motivation increased (n=16). Students stated that the chatbot encouraged their desire to learn by simplifying complex topics. There were also students who reported that learning speed was not affected (n=1) and conceptual understanding did not change (n=3). Usage-Related Limitations: Feedback regarding the limitations of the system was grouped under three main headings. The most frequently mentioned limitation was the chatbot’s potential to provide incorrect or incomplete information in some cases (n=12). In addition, technical/access issues (n=3) and inadequacies in the transfer of data containing visuals and tables (n=1) were reported by participants. Student–Faculty Interaction: Findings regarding the chatbot’s role in the teaching process demonstrate that this technology serves a complementary function (n=12) rather than replacing the faculty member. Students stated that the chatbot reduced the instructor’s workload by answering routine and repetitive questions (n=10) and that this paved the way for more qualified academic interaction with the instructor (n=10). Some students (n=2) reported that it reduced academic interaction with the instructor (**Figure 5**).

## 5 Discussion

The findings of this study suggest that a chatbot-assisted instruction approach integrated with the ARCS model in physiology education is associated with improved learning outcomes. In particular, the fact that this integrated structure does not create an additional increase in cognitive load while improving academic achievement indicates that motivational design and chatbot support can be effectively combined from a pedagogical perspective.

One of the key factors that influence students’ participation in the learning process and their learning behaviors is motivation (Pintrich, 2003). Students with high learning motivation exhibit discovery-based learning behaviors more frequently during the learning process (Martens et al., 2004). This suggests that motivation may play a critical role in the development of instructional approaches that support learning. It has been reported that the vast majority of approaches aimed at increasing student motivation naturally align with the four components of the ARCS model (Hodges, 2004). However, in disciplines with high conceptual density, such as physiology, implementing these motivational components with static materials can be challenging. Indeed, the abstract and mechanism-based nature of physiology courses can limit interest in this subject (Lavonen et al., 2021), which may reduce the effectiveness of traditional instructional approaches in sustaining student motivation. Integrating digital tools into motivation strategies has the potential to alleviate these challenges. In this context, the potential of chatbots to support personalized learning, provide instant feedback, and increase active participation in the learning process by assuming the role of an instructor or peer demonstrates that such technologies can be meaningfully integrated into instructional design from a pedagogical perspective (Kuhail et al., 2023). It is known that the integration of pedagogically grounded technologies into teaching makes a decisive difference in student learning (Alé and Arancibia, 2025). In this regard, the effects of using these technologies in education on learning processes need to be examined in detail (Chen et al., 2020).

While it is accepted that motivation affects student behavior, it is unclear whether high motivation always directly translates into academic success (Goksu and Islam Bolat, 2021; Chang et al., 2022). A meta-analysis examining the impact of the ARCS model on academic achievement reported significant heterogeneity in effect sizes among the studies (Goksu and Islam Bolat, 2021). In the current study, the most academically successful group was the ARCS+Chatbot group. The main reason for this result may be that the chatbot supports the four components of the ARCS model simultaneously and functionally. The chatbot has the potential to strengthen the “Attention” component by providing immediate responses and facilitating continuous interaction, enabling students to receive contextualized and content-specific answers to their individual questions. By offering opportunities for repetition and creating a low-risk practice environment, it may also enhance the “Relevance” component. Furthermore, through the provision of rapid and continuous feedback, the chatbot has the potential to support the “Confidence” and “Satisfaction” components. This holistic support structure may have increased the effect of applying the ARCS model with the chatbot on academic achievement and may explain why the ARCS+Chatbot group showed higher achievement compared to other groups. When interpreting these findings, it should be considered that individual variables such as students’ prior experience with AI-based tools, academic preparedness, and prior academic performance may have influenced the academic achievement differences observed in the ARCS+Chatbot group. Findings suggesting that the effect of AI-supported chatbots on academic performance may emerge through learning motivation (Caratiquit and Caratiquit, 2023) suggest that the differences in achievement observed between groups in the current study can be explained not only by a direct effect but also by potential motivational processes. However, the mediating role of motivation was not directly tested in this study. Therefore, interpretations regarding motivation should be viewed not as causal or mediational inferences, but as a literature-based theoretical interpretation of the current findings. Additionally, empirical findings indicate that the use of chatbots in English language education increases both intrinsic and extrinsic learning motivation (Ali et al., 2023) suggest that the positive patterns of learning and technology acceptance observed in the ARCS+Chatbot group in the present study may align with broader educational trends rather than being specific to this field.

While the ARCS model has been reported in the literature to optimize cognitive load, it is emphasized that this effect is sensitive to content difficulty, intervention intensity, and the structure of the learning environment (Evans et al., 2024; Cui and Fang, 2021). It has been reported that structured ARCS model design can reduce cognitive load, particularly with high-difficulty content, but this effect is not evident in more balanced or short-term learning tasks (Cui and Fang, 2021). Complementary teaching strategies such as ARCS model can increase students’ cognitive load and weaken action control when not properly structured (Astleitner and Lintner 2004). In our study, the fact that no significant difference was found in cognitive load levels between the traditional instruction, ARCS, and ARCS+Chatbot groups suggests that the ARCS model may function as a framework that supports the learning process rather than a mechanism that directly alters cognitive load. The absence of significant differences in cognitive load across groups should be interpreted with caution; because assessing cognitive load using a single-item self-report measure may have limited sensitivity in detecting subtle differences related to the multidimensional nature of cognitive load. Therefore, the observed lack of significance suggests that potential small effects may not have been detected due to the limitations of the measurement tool. Although studies directly examining the relationship between ARCS model and cognitive load are limited, the interaction between motivation and cognitive load reveals important implications for instructional design. It is noted that this relationship depends on preserving relevant cognitive load that contributes to learning and establishing a balanced structure between internal and external cognitive load (Lan, 2021). It is reported that motivation is not a variable that directly reduces cognitive load, but under appropriate instructional design conditions, it contributes to the regulation of especially external and perceived cognitive load by enabling more efficient use of cognitive resources (Evans et al., 2024). Furthermore, it has been shown that cognitive load levels do not determine learning success on their own and that their relationship with learning outcomes is sensitive to variables (Chen et al., 2009; Küçük et al., 2014). Indeed, it has also been reported that the relationship between cognitive load and behavioral intentions may emerge through motivation (Cheng, 2017).

Being in close contact with technology in general does not directly explain why students perceive a particular educational technology as more useful or easier to use (Davis et al., 1989). In this context, the variables Perceived Usefulness and Perceived Ease of Use, evaluated within the TAM in the study, were examined not only to reveal the effect of the chatbot-supported ARCS model on academic achievement but also to determine the extent to which students embraced this technology as a meaningful and functional tool in the learning process. The findings show a significant increase in perceived usefullness levels following the implementation; this may indicate that the chatbot-assisted ARCS model is perceived as a tool that contributes to the learning process and is aligned with academic goals. This increase appears to be associated with the implementation of the chatbot-supported ARCS model; however, given the absence of a comparison group, this interpretation should be considered within the context of the study design. The increase in perceived benefits within the ARCS+Chatbot group may be related to motivational processes rather than merely usability-focused processes (Lai et al., 2023). Although perceived benefits increased, perceived ease of use remained unchanged. This finding suggests that students prioritize the learning benefits of generative AI systems over their ease of use when evaluating such technologies (Le et al., 2026); a similar trend was observed for the AI- supported chatbot used in this study. Additionally, the lack of a significant difference between pre-test and post-test scores in the “Perceived Ease of Use” dimension may indicate that students viewed the chatbot system as an accessible and understandable technology from the outset. Taken together, these findings suggest that the integration of the chatbot-supported ARCS model into the learning environment does not pose a barrier in terms of ease of use, and that its contribution to learning may be perceived more clearly by students as the process progresses. This comment aligns with studies reporting that students find chatbots useful and easy to use and that previous interactions increase behavioral engagement (Hew et al., 2021), as well as findings demonstrating that chatbots have pedagogically meaningful potential in university-level health education (Kang et al., 2023).

The qualitative findings of the present study indicate that students tend to perceive the chatbot not as a replacement for the instructor, but rather as a complementary tool that supports the learning process. Students specifically stated that its speed, easy accessibility, and ability to repeat information facilitated the learning process and helped quickly resolve cognitive disconnects experienced during class. These features may highlight the chatbot’s potential to reduce the teacher’s workload and, indirectly, support in-class interaction. Similarly, findings suggesting that AI-supported chatbots can support self-regulated learning processes—such as setting individual learning goals, accessing appropriate resources, and planning and monitoring the learning process—may explain why the students in this study perceived the chatbot as an accessible and functional learning support tool (Lin, 2024). These responses suggest that students view the chatbot as a practical learning tool that simplifies complex concepts and is instantly accessible; this aligns with findings demonstrating the pedagogical contributions of conversation-based agents in education (Liu et al., 2024; Lai and Ho, 2025). However, disadvantages such as the possibility of generating incorrect or incomplete information and the limited availability of visual support were highlighted by students as notable limitations. Concerns were also expressed that excessive and uncontrolled use of the chatbot could weaken face-to-face interaction. When considered collectively, these findings—namely the increase in academic achievement in the ARCS+Chatbot group, the absence of a significant change in cognitive load levels, and the increase in perceived usefulness—suggest a possible alignment between the quantitative and qualitative results. In other words, the impact of chatbot support may have emerged not only through changes in learning as reflected in perceptual assessments, but also through experiential mechanisms that accelerate processes, enhance accessibility, and support learning. However, since these findings are based on a brief intervention of approximately three hours, they should primarily be interpreted within the context of short-term effects.

## 6 Limitations and future directions

### 6.1 Limitations of the study

- Single-center design: The fact that the study was conducted at a single institution and with a limited sample size may limit the generalizability of the findings to different educational settings and student groups.
- Design-related limitations: Although the groups were conducted in separate class sections to minimize interaction between participants, potential clustering effects cannot be entirely ruled out. This may have influenced the independence of group conditions.
- Content limitation: The fact that the teaching intervention was limited to a specific physiology topic makes it difficult to generalize about the effectiveness of chatbot-supported and motivation-based teaching approaches in different course contents.
- Short intervention duration: The relatively short duration of the teaching application did not allow for the assessment of long-term learning retention and sustainable changes in learning behaviors.
- Self-report-based measurement tools: The fact that some of the data collection tools rely on participant self-reports carries the potential for response bias and may lead to results being influenced by participant perceptions.
- Uncontrolled individual variables: Students’ individual learning characteristics and previous technology use experiences were not controlled. These variables may affect learning performance and participation levels.
- Cognitive load test: A single-item measurement approach may not fully reflect the multidimensional nature of cognitive load and may have limited sensitivity, particularly when it comes to detecting subtle differences between groups.
- TAM scale: The fact that the TAM analysis was conducted only in the ARCS+Chatbot group may limit the interpretability of the findings. Given the absence of a comparison group, the attribution of changes in perceived benefits solely to the intervention should be evaluated with caution. Furthermore, the possibility that novelty or expectation effects may have played a role in the observed changes should not be overlooked.
- Qualitative data limitations: Open-ended interview questions may have steered participants toward specific aspects of chatbot usage. This may have limited the diversity of the views obtained.
- Exploratory nature of findings: Due to the methodological limitations mentioned, the study findings cannot be considered generalizable results.

### 6.2 Recommendations for future research

- Conducting future studies with multi-center designs and larger, heterogeneous student samples may increase the external validity of findings.
- Longitudinal studies are needed to examine the long-term effects of AI-supported motivation-based instructional designs on learning retention and learning behaviors.
- The effectiveness of ARCS model-supported chatbot integration should be investigated across different disciplines, particularly in areas requiring higher-order thinking and applied problem-solving skills.
- Examining instructional design variables that shape chatbot effectiveness will contribute to the optimal use of these technologies in educational settings.

## 7 Conclusion

The integration of the ARCS motivation model with artificial intelligence-supported chatbot technology can be considered an innovative, applicable, and pedagogically meaningful teaching approach with the potential to increase academic achievement in physiology education. The findings suggest that when motivation-based instructional designs are supported by digital and interactive learning environments, students tend to engage more actively in the learning process, conceptual understanding may be strengthened, and the learning experience may become more enduring. Furthermore, it is thought that AI-supported systems can create a learning environment that is sensitive to student needs by offering individualized learning opportunities, thereby increasing the accessibility and continuity of the learning process. However, it is critically important for chatbots to be structured as complementary learning tools that support the teaching process rather than as tools that replace teaching staff in order to maintain pedagogical effectiveness. In this context, the study shows that the integration of motivation-based instructional approaches with AI-supported learning technologies can contribute to the development of innovative instructional designs, particularly in health sciences education, and provide an important theoretical and practical framework for future technology-supported learning models.

## Data Availability

The raw data supporting the conclusions of this article will be made available by the authors, without undue reservation.
