# Supplementary material for: The effect of using a chatbot integrated with the ARCS motivation model in physiology education: a mixed-methods study
Source: Front Physiol. 2026 May 28;17:1816470. doi: 10.3389/fphys.2026.1816470 (PMC13253447; doi:10.3389/fphys.2026.1816470)
Supplement: Supplementary file 1 [file Table1.docx]

**Supplementary Figure 1**

**Academic Achievement Test**

**1. Which of the following is NOT a function of the respiratory system?**

A) Producing sound

B) Contributing to the regulation of blood pH

C) Converting angiotensin I to angiotensin II

D) Protecting respiratory surfaces against dehydration and temperature changes

E) Increasing body temperature

**2. Which of the following is NOT included among the four major mechanisms by which respiration can be examined?**

A) Pulmonary ventilation

B) Gas exchange

C) Transport of oxygen and carbon dioxide

D) Neural control of respiration

E) Direct regulation of blood pressure

**3. Which structures form the part of the respiratory system where gas exchange occurs?**

A) Trachea and bronchi

B) Larynx and pharynx

C) Respiratory bronchioles and alveoli

D) Terminal bronchioles and trachea

E) Bronchi and terminal bronchioles

**4. Which of the following statements correctly describes the mechanism of normal expiration?**

A) It is achieved by strong contraction of the diaphragm

B) It occurs through upward and forward movement of the ribs

C) It is an active process requiring contraction of expiratory muscles

D) It is a passive process resulting from elastic recoil of the lungs and thorax

E) It occurs due to a decrease in alveolar pressure caused by increased lung volume

**5. Which of the following is considered the primary muscle of inspiration?**

A) Abdominal muscles

B) Internal intercostal muscles

C) Diaphragm

D) Neck muscles

E) Pectoral muscles

**6. During exercise or in conditions such as asthma where airway resistance increases, which are the main muscle groups assisting forced expiration?**

A) Diaphragm only

B) External intercostal muscles and accessory neck muscles

C) Abdominal muscles and internal intercostal muscles

D) Internal intercostal muscles only

E) Pectoral muscles and scalene muscles

**7. What is the name of the double-layered membrane that surrounds the thoracic cavity and covers the lungs?**

A) Pericardium

B) Mediastinum

C) Pleura

D) Peritoneum

E) Meninges

**8. Which of the following cell types is responsible for gas (air) exchange in the lungs?**

A) Type I alveolar cells

B) Type II alveolar cells

C) Alveolar macrophages

D) Goblet cells

E) Club (Clara) cells

**9. Calculation of minute or total ventilation is obtained by multiplying which two values?**

A) Residual volume × Respiratory rate

B) Tidal volume × Respiratory rate

C) Vital capacity × Tidal volume

D) Anatomical dead space × Respiratory rate

E) Inspiratory reserve × Expiratory reserve

**10. Which of the following terms refers to a temporary cessation of breathing?**

A) Hyperpnea

B) Dyspnea

C) Polypnea

D) Apnea

E) Eupnea

**11. What does pulmonary compliance (distensibility) refer to?**

A) The contractile force of respiratory muscles

B) The ability of the lungs to resist airway resistance

C) The ability of the lungs to expand

D) The oxygen-binding capacity of hemoglobin in blood

E) The absolute value of alveolar surface tension

**12. Which of the following substances prevents alveolar collapse by reducing surface tension in the alveoli through a detergent-like effect?**

A) Plasma

B) A mixture of lecithin and sphingomyelin (surfactant)

C) Myoglobin

D) Angiotensin I

E) Carbaminohemoglobin

**13. Compared to the systemic circulation, how is the pulmonary circulation characterized?**

A) High pressure and high resistance system

B) High pressure and low resistance system

C) Low pressure and high resistance system

D) Low pressure and low resistance system

E) A system in which systolic pressure is lower than diastolic pressure

**14. How is oxygen transported in the blood after passing from the lungs?**

A) 5% bound to hemoglobin, 95% dissolved in plasma

B) 97% bound to hemoglobin, 3% dissolved in plasma

C) 100% bound to hemoglobin

D) 50% bound to hemoglobin, 50% bound to plasma proteins

E) 80% dissolved in erythrocyte membrane, 20% dissolved in plasma

**15. In which form is the majority (60–70%) of carbon dioxide transported in the blood?**

A) Dissolved in plasma

B) As carbaminohemoglobin

C) As carbonic acid (H2CO3)

D) As bicarbonate ion (HCO3–)

E) As free CO2 gas

**16. Which type of hypoxia occurs as a result of the tissue being unable to utilize oxygen, as seen in cyanide or alcohol poisoning?**

A) Anemic hypoxia

B) Stagnant hypoxia

C) Histotoxic hypoxia

D) Hypoxic hypoxia

E) Hypercapnic hypoxia

**17. Peripheral chemoreceptors are sensitive to blood PCO2, pH, or PO2. Where are most of these receptors located?**

A) In the cerebrospinal fluid

B) In the pulmonary arteries

C) In the carotid sinus

D) In the respiratory neurons of the brainstem

E) In stretch receptors of the lungs

**18. Which of the following is NOT one of the sensory inputs that affect the respiratory centers?**

A) Inputs from lung stretch receptors sensitive to changes in lung volume

B) Inputs from chemoreceptors sensitive to blood or cerebrospinal fluid PCO2

C) Inputs resulting from pain and changes in body temperature

D) Decreased CO2 transport capacity of blood (hemoglobin concentration)

E) Impulses generated by changes in blood pressure in the aorta or carotid sinuses

**19. Write the name of the volume appropriate for the blank. The volume that cannot be measured with spirometry is …………………… volume.**

**20. In the lung volume graph below, write the name of the volume or capacity indicated by the letters A and G.**

**
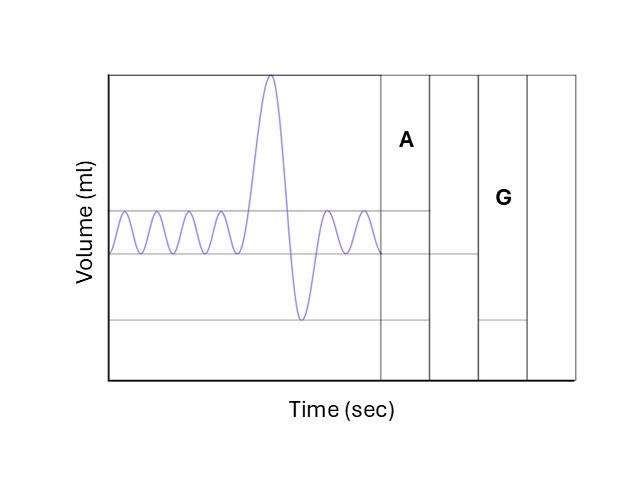
**

**A:**

**G:**

**Supplementary Figure 2**

**Cognitive Load Scale (Paas & Merriënboer, 1993)**

Please indicate the level of mental effort you invested while studying the physiology content by selecting one value on the scale from 1 (very low mental effort) to 9 (very high mental effort).

How much mental effort did you invest while studying the physiology content?

| Concepts | Very, very low | Very low | Low | Somewhat low | Neither low nor high | Somewhat high | High | Very high | Very, very high |
| --- | --- | --- | --- | --- | --- | --- | --- | --- | --- |
|  | 1 | 2 | 3 | 4 | 5 | 6 | 7 | 8 | 9 |

**Supplementary Figure 3**

**Technology Acceptance Model Scale**

Below are statements regarding the use of an artificial intelligence–supported chatbot. Please indicate the extent to which you agree with each statement by marking the appropriate option.

1 = Strongly Disagree 2 = Disagree 3 = Neutral 4 = Agree 5 = Strongly Agree

| **Perceived Usefulness** | | **1** | **2** | **3** | **4** | **5** |
| --- | --- | --- | --- | --- | --- | --- |
| 1 | **Without a chatbot, it would be difficult for me to understand the course.** |  |  |  |  |  |
| 2 | **Using a chatbot allows me to study my courses in a more controlled manner.** |  |  |  |  |  |
| 3 | **Using a chatbot increases my academic performance.** |  |  |  |  |  |
| 4 | **The chatbot meets my learning needs related to the courses.** |  |  |  |  |  |
| 5 | **Using a chatbot saves me time.** |  |  |  |  |  |
| 6 | **The chatbot enables me to complete my work more quickly.** |  |  |  |  |  |
| 7 | **The chatbot supports the critical aspects of my learning.** |  |  |  |  |  |
| 8 | **Using a chatbot enables me to comprehend more topics than I would normally be able to learn.** |  |  |  |  |  |
| 9 | **The chatbot reduces the time I spend on inefficient activities.** |  |  |  |  |  |
| 10 | **Using a chatbot improves the quality of my learning.** |  |  |  |  |  |
| 11 | **Using a chatbot improves the quality of the work I do.** |  |  |  |  |  |
| 12 | **Using a chatbot increases my productivity.** |  |  |  |  |  |
| 13 | **Using a chatbot makes studying easier for me.** |  |  |  |  |  |
| 14 | **Overall, I find the chatbot useful for studying.** |  |  |  |  |  |
| **Perceived Ease of Use** | |  |  |  |  |  |
| 1 | **I often feel confused when using the chatbot.** |  |  |  |  |  |
| 2 | **I often make mistakes when using the chatbot.** |  |  |  |  |  |
| 3 | **Using the chatbot is often frustrating.** |  |  |  |  |  |
| 4 | **I frequently need a user guide when using the chatbot.** |  |  |  |  |  |
| 5 | **Using the chatbot requires a great deal of mental effort.** |  |  |  |  |  |
| 6 | **It is easy for me to correct the errors I encounter when using the chatbot.** |  |  |  |  |  |
| 7 | **The chatbot is not flexible in terms of use; it is rigid.** |  |  |  |  |  |
| 8 | **It is easy for me to get the chatbot to do what I want.** |  |  |  |  |  |
| 9 | **The chatbot generally behaves in an unexpected way.** |  |  |  |  |  |
| 10 | **I find the chatbot inconvenient to use.** |  |  |  |  |  |
| 11 | **Using the chatbot is easy for me.** |  |  |  |  |  |
| 12 | **When using the chatbot, it is easy for me to remember how to carry out the learning process.** |  |  |  |  |  |
| 13 | **The chatbot provides helpful guidance on topics related to the lessons.** |  |  |  |  |  |
| 14 | **Overall, I find the chatbot easy to use.** |  |  |  |  |  |

**Supplementary Figure 4**

**Semı-Structured Intervıew Form**

1. Did the chatbot-supported learning environment have an effect on your learning speed and level of understanding? Can you explain your answer?
2. Did the chatbot-supported learning environment affect your motivation to learn the course?
3. What difficulties did you encounter while using the artificial intelligence–based chatbot (technical, conceptual, access-related, etc.)?
4. How do you think these tools affected student–instructor interaction?
